# Lifetime development of behavioural phenotype in the house mouse (*Mus musculus*)

**DOI:** 10.1186/1742-9994-12-S1-S17

**Published:** 2015-08-24

**Authors:** Vera Brust, Philipp M Schindler, Lars Lewejohann

**Affiliations:** 1 Behavioral Biology, University of Osnabrueck, Barbarastrasse 11, 49076 Osnabrueck, Germany

**Keywords:** behavioural phenotype, ontogeny, phenotypic plasticity, behavioural stability, life stages, prenatal, postnatal, adolescence, adulthood, post-reproductive age

## Abstract

With each trajectory taken during the ontogeny of an individual, the number of optional behavioural phenotypes that can be expressed across its life span is reduced. The initial range of phenotypic plasticity is largely determined by the genetic material/composition of the gametes whereas interacting with the given environment shapes individuals to adapt to/cope with specific demands. In mammalian species, the phenotype is shaped as the foetus grows, depending on the environment in the uterus, which in turn depends on the outer environment the mother experiences during pregnancy. After birth, a complex interaction between innate constitution and environmental conditions shapes individual lifetime trajectories, bringing about a wide range of diversity among individual subjects.

In laboratory mice inbreeding has been systematically induced in order to reduce the genetic variability between experimental subjects. In addition, within most laboratories conducting behavioural phenotyping with mice, breeding and housing conditions are highly standardised. Despite such standardisation efforts a considerable amount of variability persists in the behaviour of mice. There is good evidence that phenotypic variation is not merely random but might involve individual specific behavioural patterns consistent over time. In order to understand the mechanisms and the possible adaptive value of the maintenance of individuality we review the emergence of behavioural phenotypes over the course of the life of (laboratory) mice. We present a literature review summarizing developmental stages of behavioural development of mice along with three illustrative case studies. We conclude that the accumulation of environmental differences and experiences lead to a “mouse individuality” that becomes increasingly stable over the lifetime.

## Introduction

Reduction of phenotypic plasticity (i.e. the ability of one genotype to produce various phenotypes when exposed to different environments) is a gradual process during the ontogeny of an individual. So far it was widely accepted that the full initial range of plasticity is determined by the genetic material/composition of the gametes. Thus, in a theoretical framework which can be imagined as in Waddington's landscape model [1], there is an almost endless range of possible phenotypic outcomes for each individual. Environmental conditions experienced during the ontogeny of an individual set stage for individual trajectories and thereby successively reduce the number of options [2]. With each trajectory taken the individual adapts to the specific environmental demands. In recent years it has been shown that epigenetic modifications persist over several generations and apparently act even before fertilisation begins [3]. Hence, environmental as well as genetic and epigenetic factors begin shaping an individuals' phenotype well before birth. In mice, as in all mammalian species, phenotypes are shaped as the foetus grows, depending on the environment in the uterus, which in turn is largely dependent upon the environment the mother experiences during pregnancy. This early shaping of the phenotype of the unborn offspring has been discussed in terms of an adaptive process by which the mother shapes her progeny to suit the environment it is born into. After birth, a complex interaction between innate constitution and environmental conditions shapes the individual's phenotype and further reduces plasticity in terms of hurdles in reversing the trajectory taken. Mammals are known to change in physiology, cognitive capabilities, social dimensions, and behaviour over the course of their lifespan. In seeking to understand the ontogeny of behaviour of an individual across its lifespan, such developmental changes are of great importance. Although there is an overall gradual developmental course, different developmental phases can be identified and are indicated by distinct events or maturational changes like e.g. birth or the onset of puberty [4]. We will review these phases of life in order to provide a concise overview how developmental aspects shape the behavioural phenotype.

## A mouse's life

In laboratory mice, breeding and housing conditions are usually highly standardised, facilitating a consistent and reliable environment. Additionally, inbreeding has been systematically induced in order to reduce the genetic variability between experimental subjects [5]. These standardisation efforts, however, have not led to one standard behavioural phenotype, i.e. behavioural reactions repeatable over time and similar in each mouse. Instead, considerable variability has been found in the behaviour of laboratory mice [6]. These findings indicate that it is virtually impossible to control for all potential developmental impacts even under standardised laboratory conditions. Given these facts, a detailed analysis of the developmental stages and possible onsets of diversification of behavioural phenotypes is a timely endeavour to be addressed in this model species. A quick PubMed query [7] reveals more than 11.000 publications using the searchwords “mice”, “development”, and “behaviour”.

Interestingly, in modern biomedical research individuals are usually tested only in a single short term experiment and disposed directly thereafter. Mice are easily available by commercial breeders at any age class and previous experiments are well known to influence the outcome of future testing [8]. Consequently, the usage of new animals in each test has clear advantages that may have led to this prevalent use of mice as “disposable goods” in science. However, one consequence of this approach is that long term studies, tracking the development of individuals over their whole lifetime, have been widely neglected. Accordingly, an extended PubMed search including an additional term to filter for at least one of three terms “lifetime”, “long-term” or “longitudinal” ([9]; 2015/03/15), narrows the number of papers available down to 89. This search query reveals that the lion's share of information on behavioural development in the mouse is highly fragmented. This is not only true for research papers, but also for previous reviews, which deal either with behavioural development in tightly restricted periods of life (e.g. [10]) or focus on single causes of alterations in behaviour over longer time periods (e.g. [11]).

## Phases of life in mice

In this review, we aim at putting the available pieces together to take a first step to reveal the full picture of behavioural development. In doing so, we will embrace all five main stages of mouse development, i.e. the prenatal phase, the early postnatal phase, adolescence, adulthood, and the post-reproductive phase. Each phase description will consist of a review of the present literature record. We will provide three additional case studies using our own data to illustrate important concepts relevant to the developmental phases. For the sake of conciseness, we will focus our review on proximate mechanisms and only briefly refer to possible adaptive values. For evolutionary aspects of behavioural development we encourage reading accompanying papers in this issue by [12] and [13].

**Figure 1:**
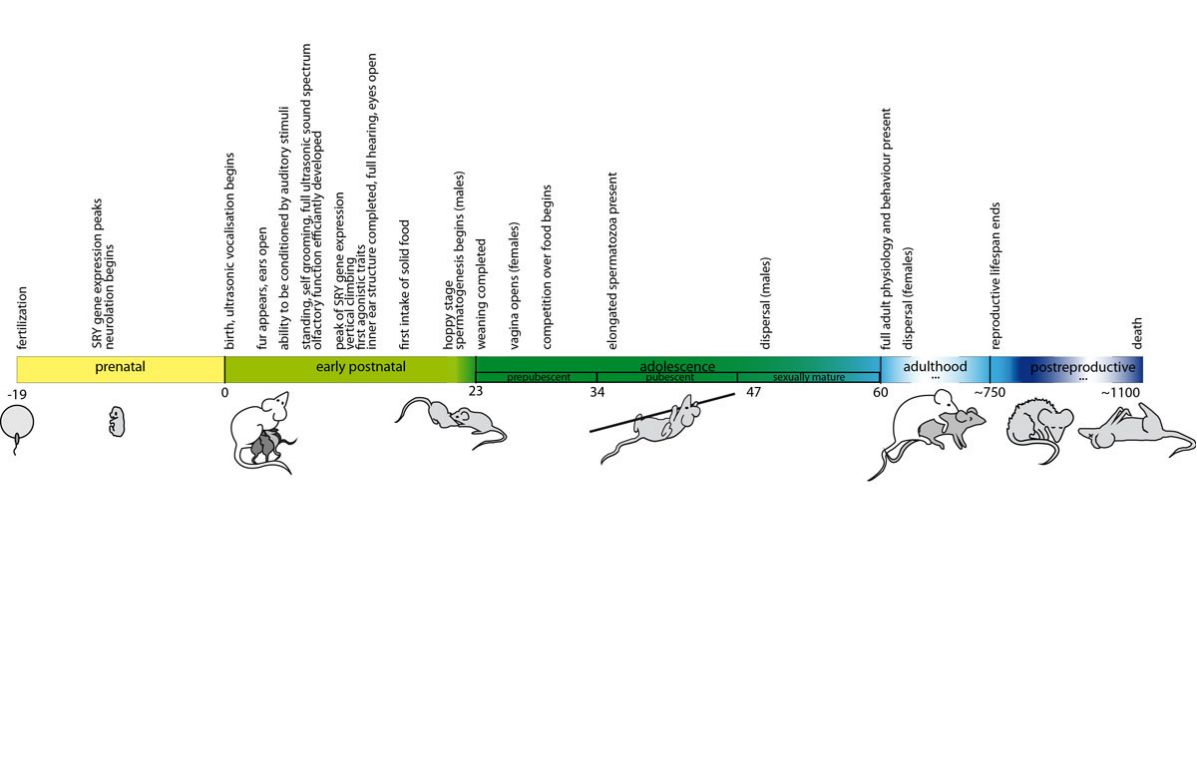
**Development of physiology and behaviour over the lifetime of a mouse.** Events are indicated at the mean time point of their occurrence according to the literature record including various non-genetically modified strains of mice. Detailed information on the expression of single traits can be found in the corresponding text sections.

The developing foetus underlies daily changes during the process of prenatal ontogeny, which is generally highly predictable in mice [14]. Still, the exact timing of developmental events and consequently the exact end of the prenatal phase can vary, leading to a delayed or advanced onset of the postnatal phase in a range of about one day [15]. After being born the variation with regard to the exact timing of developmental milestones expands to several days and even months as the mice grow older. Different strains of inbred mice do further extend or shorten certain life periods or have shifted distinct developmental events (e.g. [16-19]). In addition, distinct environmental cues might delay or increase the speed of maturation [20]. Thus, the time of the onset and end of the following phases described here can differ up to several days depending on the criteria and strain of mice used. Be this as it may, in principle all events are harmoniously coordinated and occur in the same order in every individual [21]. Within each phase there can be salient developmental moments (e.g. SRY gene expression leading to sexual differentiation [22]) with special impact on behavioural phenotypes leading to a non-linear development. A detailed description of the five stages of development and important events therein follows below; for an overview see Fig. 1. Male and female mice differ in development over the course of their lives. Whenever major sex differences were obvious from the literature we state this. However, for a more detailed view of how sex related personality differences emerge see [23].

## The prenatal phase

In recent years it has been shown that environmental factors mediated by epigenetic influences shape the behavioural phenotype [3,24]. For example, the descendant F1 and F2 generations of olfactory fear conditioned C57BL/6J male mice showed a significantly increased sensitivity to odour cues their fathers or grandfathers were conditioned to [25].

After conception a single cell develops to give rise to a complex, multicellular organism over a period of 19 days. During this period the fertilised egg divides and a variety of cell types, specialised tissues, and organs are shaped. Processes during embryogenesis include coordinated cell division, cell specialisation, cell migration, and genetically programmed cell death [26]. Of special interest for any behavioural development are processes following the neurulation which begins around embryonic day 8.5 [27] with the closure of the neural tube. The neural tube develops into the spinal cord and the brain, orchestrated by coordinated and precisely timed gene expression of thousands of genes [28]. During the maturation of neuronal tissues the brain circuits are highly plastic. In male mice at embryonic day 11.5 the SRY gene transcription peaks [29] and initiates sexual differentiation with the induction of Leydig cells secreting testosterone. Interestingly, there is an inter-uterine transfer of testosterone between foetuses. Thereby individuals are affected by the number of neighbouring males leading to developmental differences in physiology and behaviour later in life [30]. Generally, it is assumed that changes in the uterine environment are capable to exert long-lasting changes in behaviour and physiology [31-34]. Already during prenatal development, motor-sensory and cognitive behaviours can be observed in rodents [35,36]. Testing learning abilities *in utero* is challenging and was conducted during the last day of uterine life in rats in a taste/odour aversion learning task [36]. In mice, possibly due to size limitations, tasks investigating early learning have been conducted more focused on the postnatal phase, with successful conditioning as early as PND 3 [37].

It has been suggested that any alterations in behavioural phenotype that are linked to specific experiences in early pre- and postnatal life represent adaptive maternal effects, allowing mothers to adjust their offspring to the prevailing environment [38,39]. Basically, the mother has a limited number of options to raise offspring perfectly adapted to the future environment: by means of female choice the mother can choose a male that is most promising to father high quality offspring. Indeed it could be shown that offspring resulting from preferred mating partners had a significantly higher fitness than offspring from non-preferred males [40]. However, in wild living house mice and especially in breeding colonies of laboratory mice female choice might be limited or restricted to the one male available for mating. After mating, the mother might reduce [41] or even stop any investment into the current offspring either by terminating pregnancy [42] or abandoning or killing her young immediately after giving birth [43]. These phenomena are often observed in breeding colonies of laboratory mice. Noteworthy, it has been shown by Hilda Bruce and colleagues that these processes can indeed be adaptive: males taking over a territory often kill the offspring sired by the former territory holder. By terminating pregnancy females might increase their lifetime fitness by stopping an investment into a litter that is doomed in favour of a litter sired by the new territory holder [42], “Bruce effect”.

If the litter is carried to term, the young will have to deal with the environment they are born in, be it good or adverse. Naturally, a beneficial environment (e.g. food abundance, low risk of predation and infant killing, stable and predictable social conditions) would be completely different compared to a challenging adverse environment (e.g. food shortage, high predation risk, social instability). As the genetic basis has been set before by female choice (or the lack of any choice), only modifications by means of shaping the developing foetus can further increase the chance of survival. Since during gestation the mother is the only link between her offspring and the current environment, it is likely that such effects are mediated by intra-uterine mechanisms (e.g. [38]). Special emphasis regarding the mechanisms has been laid on stress and stress hormones which are capable of crossing the placenta and thus directly affect intra-uterine development. Stress hormone levels are increased in pregnant females that encounter adverse environmental conditions. In order to experimentally model such adversity, pregnant females have been exposed to a variety of stressors such as daily handling, repeated saline injections, constant light, noise, forced swimming, olfactory cues of unknown males, or repeated restraint [39,44-51].

Whereas in rats these procedures reliably cause increased anxiety-like behaviour, decreased locomotion and altered hypothalamic–pituitary–adrenal (HPA) axis regulation [31,39,46,47,51-53], such treatments do not seem to affect the offspring in a consistent way in mice. In some studies anxiety-related behaviour was reduced later in life [54] while in other studies increased anxiety [55,56] or no change in anxiety-related behaviour was found [39]. The exposure to olfactory cues of unknown males (signalling the danger of infant killing)is thought to be of ecological relevance, while for example, saline injections would not occur under natural conditions. However, there is no clear indication that the nature of the stressor is related to a predictable outcome [39]. In addition, adverse or beneficial conditions experienced by the mother can have direct effects on the foetus during pregnancy or influence maternal care behaviour after birth. Therefore it cannot always be clearly distinguished if any effects of experiences the mother made prior to giving birth on her offspring are induced during pregnancy or mediated through postnatal maternal behaviour [39].

## Case study: long term prenatal effects

**Figure 2:**
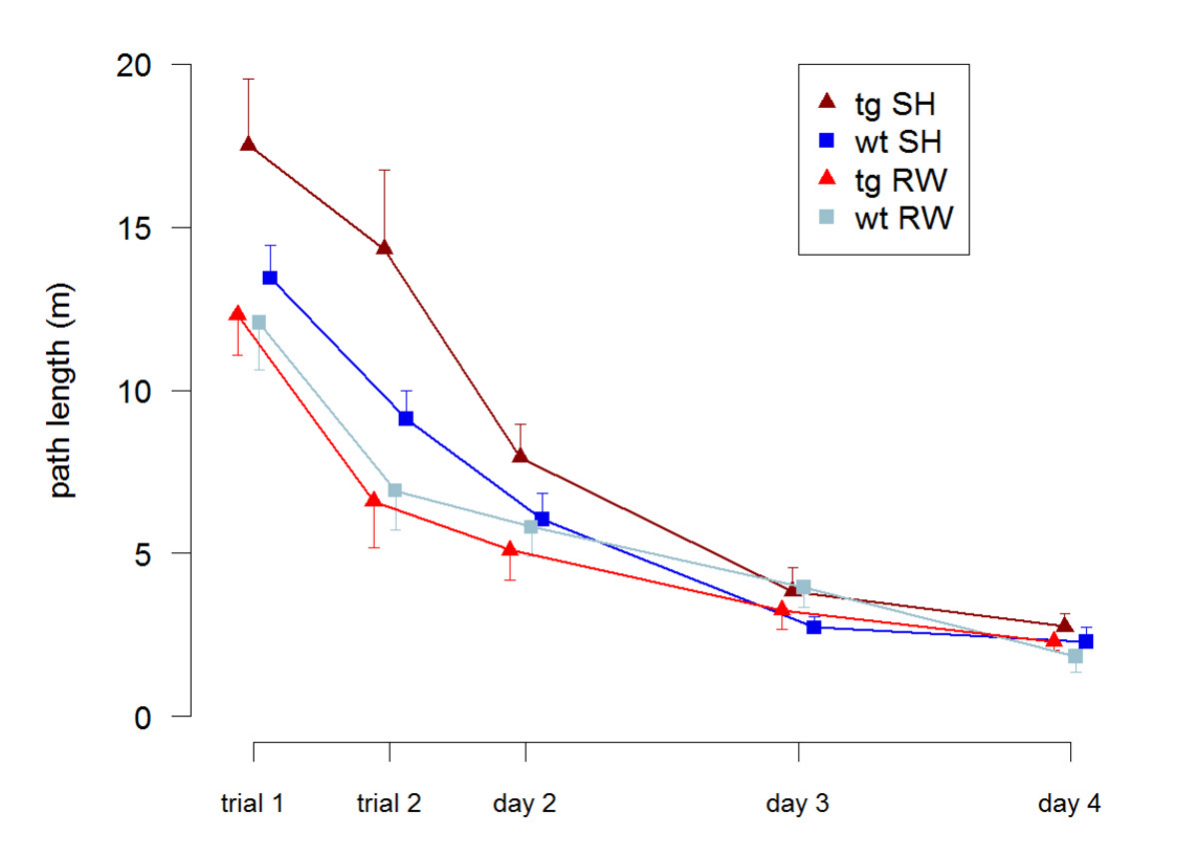
**Enhanced spatial memory in offspring of mothers with physical activity during pregnancy.** Transgenic (tg) and wild type (wt) mice modelling Alzheimer's disease were tested for spatial memory in a Barnes maze. The mothers of half of the animals were physically active by using a running wheel (RW) during pregnancy while the other mothers lived in standard housing (SH) conditions without access to a running wheel. None of the mice tested as adults at an age of 136 days had access to a running wheel themselves. Offspring of running mothers performed significantly better in the test, indicating a long lasting effect of environmental conditions during pregnancy on cognitive behaviour in adult offspring. Figure redrawn after [103].

Apart from prenatal stress there is increasing evidence that physical activity of the mother during pregnancy impacts on behaviour later in life. It has been shown that wheel-running during pregnancy increases neurogenesis in preadolescent offspring. It was thus hypothesised that such a cognitive enhancement might persist in adulthood [57]. Especially in the light of neurodegenerative diseases, such as Alzheimer's disease, an enhancement of cognitive capacities might even serve as a cognitive reserve allowing for better coping with the neurodegenerative challenges of the adult brain. Figure 2 shows data from a study conducted with a transgenic model of Alzheimer's disease [58]. In brief, pregnant dams were divided into two groups housed alone either in standard cages (37 x 21 x 15 cm) or in cages of the same size additionally providing a running wheel. The female offspring were housed without access to running wheels. Four groups of mice differed by genotype (wt: wildtype, tg: transgenic) and conditions the mothers experienced during pregnancy (SH: standard housing, RW: running wheel) were tested for spatial memory on a Barnes maze. The test apparatus is a circular platform of 1m diameter and has 12 holes drilled equally spaced near the edge. While 11 of the holes are blind ended, one hole is connected via a tunnel to the home cage placed below the platform. Mice were tested twice a day on four consecutive days and a trial-wise decline in the path length covered on the maze served as a measure for spatial memory. An ANOVA revealed a significant effect of genotype (F_1,48_=4.54, p=0.038) and condition the mothers experienced during pregnancy (F_1,48_=5.9, p=0.019) on spatial learning. Post hoc analysis indicated that these differences were mainly due to tgRW mice showing enhanced performance compared with tgSH mice (t-test, t_20_=3.2, p=0.005). The fact that learning enhancement was most pronounced in transgenic mice carrying the neuropathological burden of plaques in their brains renders it tempting to speculate that neuroprotective mechanisms were enhanced by physical exercise of the mother. Overall this case study demonstrates that environmental effects experienced by the mother during pregnancy are potentially carried over to the unborn offspring and significantly influence their behaviour as adults.

## The early postnatal phase

Being born implies the most dramatic environmental change during the life of a mouse. Temperature and nutrition which have been constantly supplied by the mother are now variable and more or less unpredictable. In addition, struggle for livelihood comprises interacting with siblings who are competing for the same resources. Although mouse pups are altricial and are thus born in a highly immature, blind and deaf state, they do have whiskers and the ability to process tactile as well as olfactory and thermal cues on their first day of life [59]. The hair begins appearing around three days of age but thermoregulation continues to be largely dependent upon ambient temperature as well as the quality of the nest, maternal care, and position within the litter [60,61]. Learning tasks have been conducted successfully with mice at PND 3 although full retention of learning after 24 hours could only be detected in mice from PND 9 onwards [37]. Ultrasonic vocalisation eliciting maternal care begins after birth and increases in intensity until around PND 8 [62]. The ears open on PND 3 [63] and the ability to be conditioned to auditory cues is present as early as on PND 4 [15]. Still, the complete development of the inner ear structures takes more time and is completed at PND 13 [63]. The eyes open around that time, too, in the range of PND 11-13 [15,63]. Until around day 16, mice pups are fed by nursing only. Nursing continues until an age of about 22 days and is successively complemented by solid food. The digestive system of new-born mice is also dependent on maternal care and pups produce ultrasonic wriggling calls to demand licking of the trunk by the mother for stimulating digestion, urination and defecation [64]. From PND 0 onwards a rapid development of behavioural abilities and reflexes begins. First complex motor abilities like forelimb grasping, placing of hindlimbs, standing, and self-grooming develop continuously over the first six days of life [16]. First agonistic traits, i.e. biting and defensive posture can be elicited between PND 12-14. With full sensory perception available, young mice following PND 14 are most of their time engaged in exploratory and investigatory behaviour. Along with developing motor abilities the pups begin to leave the nest and explore the surroundings. As a consequence, affiliative behaviour towards the nestmates, like huddling, decreases while exploration and jumping behaviour increases [65].

On PND 15 a behavioural phenomenon is observable which is known as ‘hoppy’ or ‘popcorn’ stage and is manifested in vigorous jumping [14] based on synchronous contraction of fore and hind limb extensors [66]. This behaviour is shown with or without observable disturbing stimuli. In the absence of any noticeable stimuli, running and jumping can be considered solitary play behaviour [67]. If this behaviour is shown in response to external stimuli, it was proposed to be adaptive for avoiding predators. As sensory perception is not entirely completed, young mice are unable to discriminate between harmful and harmless stimuli. Therefore jumping behaviour serves as an alternative response to predators until optimal adult strategies that would require advanced physiological and behavioural skills are developed [15,66,68,69]. The stage peaks at around PND 20 showing an inverted u-shaped curve over the early postnatal period [65] and according to Scott & Williams [15] the hoppy-phase ends at PND 25.

Towards the end of the early prenatal phase the peak in exploratory tendency seems to have already passed, i.e. [70] found B6129SF1/J wild type mice to spend less time with an unknown object at PND 24 compared to PND 22. The early postnatal period ends about PND 21-25 with the completion of weaning and thus independence from the mother [21]. The maturation to nutritional and behavioural independence is a change in the life of young mice that marks one of the focal points in development [71]. Weaned mice are, however, still small in size and not fully sexually differentiated.

Differences in maternal care can lead to epigenetic changes during this phase of early development which can persist through life [72,73]. Tsuda et al. [71] demonstrate that stress caused by maternal separation during this life stage (2 weeks after birth) reduces plasma testosterone levels, decreases arginine-vasopressin and increases oxytocin immuno reactivity in the paraventricular nucleus of males. Such hormonal changes can lead to long term behavioural changes like an increase in aggressiveness of adolescent males. Thus, the age at which experimental subjects are separated from their mother can have great influences on a variety of behavioural traits later in life as has been shown in rats [74] as well as in mice [65].

## Adolescence

Adolescence is the developmental transition from childhood to adulthood. During this phase a multitude of hormonal and behavioural changes as well as alterations in neurobiological structures occur together with a substantial remodelling of cortical and limbic circuits [75]. This variety of changes leads to cognitive, emotional, social, and sexual maturation [76,77]. The temporal boundaries of adolescence in rodents, the exact beginning and the end, are not precisely defined. Several authors, however, see it roughly as the range from weaning (PND 22-25) to adulthood (PND 55-65). The period of adolescence can be subdivided into three intervals, **early-** (prepubescent, PND 22-34), **mid-** (pubescent, PND 35-47) and **late adolescence** (sexually mature, PND 48-60) [78,79] which will be characterized in more detail in the following subsections.

Generally, the phase of adolescence is characterised by behaviour and physiology that differs substantially from adulthood, e.g. there is a general trend of increased open field activity throughout all three stages of adolescence well into adulthood [17,70,80,81]. Increased food consumption during this phase is accompanied by high energy expenditure [17]. Furthermore, stressors cause greater behavioural changes than in previous or following periods of life. For example, four week old mice exposed to social or restraint stress showed an increase in anxiety measured in an elevated plus maze. In contrast, anxiety was unchanged in stressed versus unstressed individuals tested at eight weeks of age [82]. One reason for this higher reactivity to stressful events might be maturational changes in the hypothalamic–pituitary–adrenal (HPA) axis [83] which is most likely more sensitive to stress during adolescence [84]. Vulnerability to stress is thus linked to the state of sexual maturity [82].

The stage of **early adolescence** starts at weaning and is characterised by the onset of sexual maturation and an increase in growth hormones which peak at PND 28-30 [85]. In female mice, the vagina is closed at birth and opens at around PND 26. In male mice, spermatogenesis starts around day 21 with elongated spermatozoa present at around day 35 [86]. Characteristic behavioural and physiological patterns like greater exploration, lower anxiety and a lowered stress reactivity [87] sum up to an increased risk-taking behaviour shortly after weaning when mice begin to explore the surrounding area [88]. These traits go along with the start of an increase in activity between PND 28 and 42 in C57BL/6J mice. Aggressive behaviour is further developed beginning with competition over food at this stage [15]. Early adolescent male mice which were subjected to social stress by exposure to an isolated adult male were more affected in terms of decreased food intake, reduced growth rate, and anxiety-related plus-maze behaviour than their late adolescent conspecifics.

During **mid-adolescence,** mice become fully fertile [89]. Sexual maturity, however, precedes behavioural maturity [16]. Pheromone production in male mice is detectable in this phase of puberty [90] and elevated secretion of gonadal steroid hormones occurs [76]. Additionally, male mice begin to display severe conspecific-related aggression, although aggressive behaviour per se is not observed in all individuals before late adolescence [71].

In **late adolescence** mice start to disperse, i.e. dispersal in males is around PND 49 and in females around PND 71 [91]. Males are generally more likely to disperse than females although such a sex bias was not found in all studies [92]. Going along with this, agonistic behaviours are further developed coinciding with social encounters with unfamiliar conspecifics under natural conditions [93]. Social interest measured as time spent near an unfamiliar mouse, however, decreases slightly compared to the phase of early adolescence [94].

Experiences throughout adolescence also have profound effects on behaviour later in life. Mice exposed to chronic social stress between PND 28-77 showed increased anxiety-like behaviour in adulthood [95]. Such stressful experiences may induce serious changes in metabolism which can lead to impairments of hippocampus-dependent cognitive function or alterations of body fat distribution in adulthood involved in metabolic diseases [96,97].

Regarding anxiety measured in the open field or elevated plus maze, prepubescent mice (PND 24) exhibit more fearful behaviour than adult mice (PND 75) [98]. Locomotor behaviour in the open field was found to increase from the early and mid-adolescent phase to adulthood [17,80]. In contrast, Macri et al. [88] describe that mice of both sexes are more explorative and less anxious at PND 48 compared with adult mice at PND 61. Johnson & Wilbrecht [99] were able to demonstrate that adolescent mice (PND 26-28) often exhibit a highly flexible behaviour compared to adults (PND 60-70) in a multiple choice reversal learning test. In line with these findings are observations of increased risk taking [78] and sensitivity to drugs and alcohol consumption [79,80] during adolescence. Overall, changes in exploration, risk taking behaviour, and behavioural flexibility reflect the preparation for becoming independent [77].

## Case study: emergence of predictable individual behavioural phenotypes

**Figure 3:**
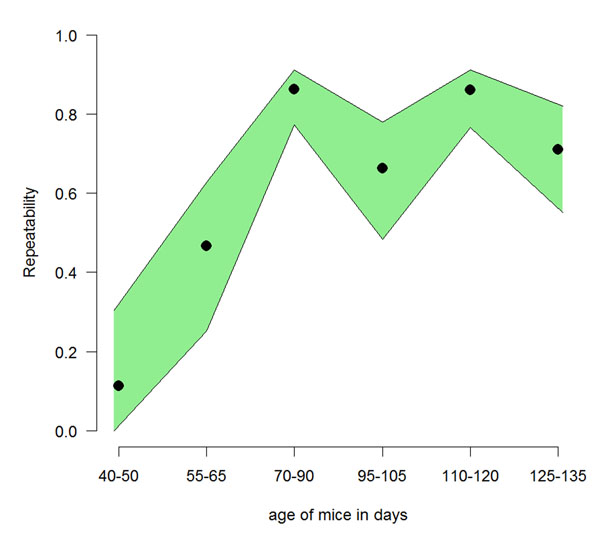
**Repeatability of activity measures increases with age.** 40 female mice (strain C57BL/6) were housed in a semi naturalistic environment and automatically tracked using RFID transponders and 20 Ring antennas [103,104]. Activity was measured from PND 35 (mid adolescence) to PND 125 (adulthood) by total antenna contacts measured in bouts of 5 days. The figure shows the repeatabilites taken from generalised linear mixed models (GLMM) including age at testing as a fixed and animal ID as a random factor. Each repeatability measure is calculated over three adjacent measurements of activity. The grouping of data is indicated on the x-axis. In green, the 95% confidence intervals of the repeatabilities are given. With the exception of the very first repeatability measurement taken between day 35 and 45, all repeatabilities are significant. As a general trend, the older the animals grew, the better the predictability for their future activity got.

As outlined above, adolescence is often described as a phase of life allowing for adjustments and optimisation of behaviour. Alterations in neurobiological structures and hormonal milieu underlie plasticity during behavioural development. Consequently, it is hypothesised that behavioural patterns shown in adolescence are less repeatable and thus also less predictive of future behavioural phenotypes. In line with this hypothesis are empirical data from other species including humans [100,101] as well as model predictions indicating that the “rate of change of behaviour is higher early in ontogeny than later in ontogeny” [102]. To illustrate this effect in mice, we return to data that were obtained in a longitudinal study [103,104]. We reanalysed the data and tested whether or not stable behavioural activity patterns emerge with age. The analysis comprises data from 40 female mice that were obtained from a commercial breeder at the age of 28 days. The mice were individually marked with subcutaneous implanted RFID transponders and from PND 35 to PND 125 they lived in a large semi-naturalistic enclosure (SNE). 20 RFID-antennas were placed at strategically chosen spots within the SNE in order to measure activity and spatial distribution for each individual. For the analysis of repeatability and predictability we summarised activity for 5-day bouts measured as total antenna visits within that time frame. Afterwards we calculated repeatabilities using generalised linear mixed models (GLMM) [105]. In order to show changes in repeatabilities over time, we split the data up in six blocks of three adjacent measurements each and calculated separate models for each block (Figure 3). As a general pattern the repeatability increases with age demonstrating an increase in behavioural stability based on extensive longitudinal measures of activity.

## Adulthood

In adulthood sexual maturity is accompanied by behavioural maturity, i.e., mice are capable of mating and successfully siring and raising offspring. Although it might be debatable whether or not an individual mouse at PND 55-65 is to be considered as early adult or as being in late adolescence, we adhere to defining adulthood as being able to sire offspring successfully. In female mice earliest mating starts around PND 35, at PND 50 half of the female mice paired with a male were mated and 100% were mated at PND 60 [106]. In male mice the balanopreputial-separation, that allows pheromonal communication, is often used as an external indicator for completed puberty and first sign of adulthood. However, the balanopreputial-separation is usually detectable as early as around PND 30 [107], which is long before mating attempts can be observed and thus falls clearly into adolescence. In general pheromonal communication is correlated with rank of male mice with subdominant adults depositing more scent marks than juveniles but less than dominant adult male mice [108]. Thus, scent marking allows priming to avoid costly aggressive interactions [109]. Male mice of the strain C57 BL/6J begin to mount at about PND 42 and SEC/IReJ males at about PND 50. Not only strain differences, but also the presence of other adult females or males can significantly bring forward or delay sexual maturation [110]. Around PND 60 the intratesticular testosterone levels of males reach adult levels [111]. Thus, commonly PND 60 is defined as the onset of adulthood where male and female mice possess a fully developed body as well as the full behavioural repertoire of the species. Behavioural changes within the adult phase seem to happen more or less continuously and no apparent developmental physiological changes are present in healthy adult mice. Activity measured in a motor activity chamber [112,113] as well as anxiety [113] and exploratory tendencies [114] seem to decrease slowly and in a more or less linear fashion. Unfortunately, statistical proof for these observed effects is rare due to a lack of tests on repeatability explicitly done with data of adult mice, although the raw data are often available (e.g. [112,115]). The general trends, however, seem to be in line with statistically confirmed findings of Marquette and Schneider [116]. They found vitality of mice in general to decline over the period of 2 to 24 months of age regardless of individual differences in experience. Notwithstanding this decline, the authors also report that activity in the open field remains stable in the same individuals within this time period. The overall decrease in performance might be due to deterioration of neuronal mediators as well as a reduction of muscles and muscle flexibility. In addition, divergent results found in different traits and testing setups might be due to differences in relative importance of changes in brain and physiology [116]. Good evidence for individual stability in performance comes from [117], who found female outbred mice (strain RjHan:NMR1) to remain stable in their anxiety-related behaviour measured in an elevated plus-maze at PND 90 and PND 135, regardless of changes in their reproductive state. In inbred mice behavioural stability was demonstrated in a study [6] by showing that male mice (strain C57BL/6N) were stable in their anxiety-related behaviour measured in an elevated plus-maze around PND 60 and PND 90. Interestingly, both studies report behavioural consistencies despite considerable inter-individual variability. Possibly some of the variability was experimentally induced by either including two groups of females with different breeding experiences [117] or different subgroups of mice that were housed in either stable or instable social groups [6].

## Case study: variability and repeatability

**Figure 4:**
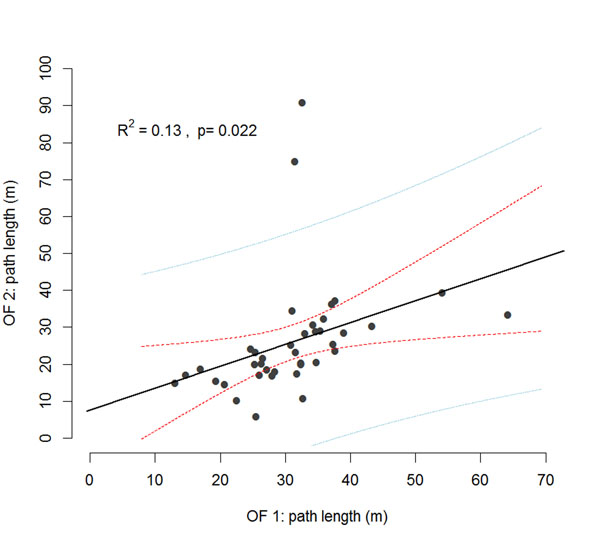
**Persistent individual differences despite standardisation.** 40 female mice of the inbred strain C57BL/6 were kept in strictly standardised conditions and tested at PND 85 and PND 120 in an open field (OF) test for locomotor behaviour. Despite genetic and environmental standardisation considerable individual differences emerged in both tests. Pearson's product-moment correlation analysis revealed a significant correlation between the different tests explaining 13% of the variance. Additionally to the regression line (black) the 95% confidence band (inner red lines) and the 95% prediction band (outer blue lines) are drawn.

In order to demonstrate that stable individual differences in behavioural patterns can emerge regardless of genetic and environmental standardisation 40 female inbred mice (strain C57BL/6N) were bought from a commercial breeder and delivered at an age of 28 days. All mice were kept following a strict standardisation regime including: light-dark cycle, lighting conditions, room temperature, air humidity, cage type and material, bedding, diet and water, testing time, testing order, testing protocol, animal caretaker, experimenter, and cage-cleaning time. The mice were tested at an age of PND 85 and PND 120 in an 80 by 80 cm open field test (OF) measuring locomotor activity for 5 minutes. Path lengths covered in the respective tests were correlated using Pearsons product-moment correlation and the coefficient of determination (R^2^) was calculated. The results shown in Figure 4 (redrawn from [118]) demonstrate that there is a considerable variation in locomotor activity in both of the OF tests despite using inbred mice and all efforts of laboratory environment standardisation. The tests were significantly correlated (t = 2.39, p=0.022, R^2^=0.13) indicating that a substantial part of the data can be explained by non-random individual consistency. This case study demonstrates that despite high efforts of standardisation a notable amount of variability persists. However, the variability is not merely random but reflects behavioural patterns which are consistent over time.

## Post-reproductive phase

In female mice the last possible parturition after which no more offspring are born can be seen as the onset of the post-reproductive phase. Cessation of male mouse fertility correspondingly is indicated by the inability to sire further offspring, although spermatozoa might still be produced at least in low numbers [119]. Wild house mice are known to be fertile until they reach an age of about 700 days. The fertile period was found to be significantly reduced to 570 days when wild mice were inbred for one generation [120]. The length of the post-reproductive phase is not only determined by the end of fertility, but naturally also by the age at which the animals die. Substantial variation in lifespan has been found between different strains of mice. While surprisingly little is known about ageing in wild mice [121], the mean lifespan of different inbred strains is well documented, and according to [122,123], ages between 256 (AKR/J), 718 (DBA/2J), and 914 (C57BL/6J) days in females and 272 (AKR/J), 741 (NZB/B1NJ), 901 (C57BL/6J) in males are common. Ageing in general is also dependent upon life history experiences. For example stress was shown to significantly reduce telomeric lengths and is therefore suspected to decrease life expectancy [124].

However, regardless of the environmentally induced individual differences as well as the genetically anchored between-strain differences, there is a considerable time span between the loss of reproductive ability and death. This time span has been estimated to be 204±32 days in 29 female and 83±29 days in 25 male F1 hybrids of C57BL6/Jlco female and CBA/Jlco male mice [125]. Over the course of the post-reproductive period the central nervous system alters its structure in accordance with behavioural changes. The cerebellum and hippocampus, for example, are impaired in electrophysiological processes and comprise altered synapses or reduced cell numbers. Such changes lead to a declining functionality, consistent with behavioural changes in humans and other animals (e.g. [122,126-130]).

In mice, studies that recorded exploratory behaviour in the open field measured as surface units crossed per time interval found a decrease in the behaviour when comparing adult mice with mice at an age of at least 22.5 months [114,131]. Along the same line, a comparison of exploration behaviour in two consecutive trials in a hole board test revealed an increase of exploration when tested in 5 months old adults. On the other hand, when the same test was conducted with aged mice (28 months), decreased exploration was found. These age-related changes in exploration behaviour have additionally been linked to changes in the hippocampal serotonergic system and are one physiological change contributing to an alteration of exploration in mice with age [132]. Generally spoken, the explorative tendencies of mice seem to decrease with age, although Brennan et al. [132] did not find these differences between 4 and 24 month old male mice of the A/J strain. Besides exploration, activity decreases in ageing individuals. As reviewed in detail in Lhotellier and Cohen-Salmon [122], ageing in mice is accompanied by a decline in motor capability comparable to motor disorders that have been found to appear along with human ageing. A decline in activity has been found across all strains of mice but might vary in its onset between strains. A decrease in exploration and locomotion is assumed to be a key factor underlying a number of age dependant performance decreases in other contexts, e.g. in cognitive performance [133].

Regarding cognition, the ability to learn is greater in adult mice compared to senescent individuals. Furthermore adults compared with aged mice do have a stronger spatial memory (e.g. [119,134]). Age linked neuronal changes, like the loss of somatostatin-positive interneuron integrity or alterations in the hippocampal serotonergic system lead to this decline in cognitive performance with age [119,132,134,135]. However, impaired cognitive abilities also appear to be an averaging phenomenon, induced by single individuals that drop in performance while many others seem not to be affected at all [134]. This indicates that there is huge individual phenotypic variability in neuronal changes during senescence.

Given that motivation as well as exploratory tendency is required to solve most cognitively challenging tasks, alterations in these traits would most likely lead to changes in cognitive performance. While exploration is known to decrease in the post-reproductive period, motivational changes are less well studied. Male C57BL6 mice at an age of 13 months do not decline in motivation to receive a food reward compared to 6 month old animals [136], but should also not yet have entered the post-reproductive phase. Shifts in motivational state with older age in mice are not reported and consequently the question whether motivational changes might also account for the post-reproductive decline in cognitive abilities remains unanswered.

## Behavioural stability

It becomes obvious from the literature reviewed above that especially during the first three phases of life the mere number of physiological, environmental and social challenges shape and alter the behavioural phenotype dramatically. Thus it is reasonable to expect that behaviour remains relatively flexible, at least during the early period of development. This, however, must not necessarily result in lower repeatability of behavioural measures during early phases of life especially when environmental conditions stay stable between tests. Adulthood can generally be characterised as a phase of higher stability with regard to physiology and behaviour. Especially with regard to social dynamics, stable physiology and behaviour may set the stage for a self-perpetuating system with predictable social interactions resulting in stable behavioural patterns and stable physiology. Still, salient lifetime events like changes in reproductive state, mating opportunities, illness, injury, and social hierarchies contribute to variability during this phase of life. The social organisation of rodents is generally dynamic and can change, for example, with variation in resource availability [137]. Changes in behaviour can be experimentally induced by changes in the social environment, i.e. isolation of mice or their introduction to new social environments. Both paradigms are stressful and lead to a decrease in locomotor activity and exploration as well as to an increase in anxiety-like behaviour in adults tested shortly after the experimental manipulation [138,139]. In line with the assumption that behaviour is more stable in adulthood compared to the previous phases of life, Bartolomucci et al. [140] found that a change in group composition at adult age did not affect behaviour compared to that of a control group that stayed with the sibling group they were raised in, while a change in group level composition during adolescence altered the behaviour of male mice. Additionally, there was no effect on long term stability of behaviour in lactating compared to non-lactating females in an elevated plus maze. This suggests that the reproductive state may not influence the stability of these behavioural traits [117]. Hence, except for minor (and possibly temporary) variations, behaviour remains relatively stable during the phase of adulthood. From an evolutionary perspective this might be a reasonable strategy to refrain from wasting energy on readjusting phenotypes as long as the chosen niche still fits.

In a recent paper [34] male mice were exposed to beneficial (communal nesting) or adverse (exposure to soiled bedding from unfamiliar males) environments during their early postnatal phase of life. Later, in mid-adolescence (37 PND), these mice were either experiencing a beneficial (housed with a female) or adverse social situation (confronted with an adult male). Mice that at an early phase of life had beneficial experiences followed by adversities at a later period of life show decreased anxiety-like behaviour compared to mice that only had beneficial conditions and mice that grew up under adversities. This shows that life history events are not simply additive in bringing about distinct adult behaviour but interact in a complex way. To explore these interaction effects and their consequences by conducting longitudinal studies will be an exciting challenge for future research.

While some studies do analyse behavioural traits repeatedly over a course of several months [136,141,142], to our knowledge no available study covers behavioural development over the full life cycle of the mouse. Additionally, the vast majority of behavioural studies in mice is conducted with individuals older than 40 days. Also in other animals, long term data on behavioural development are largely lacking. From long term studies in humans, however, we know that stability of dispositional traits increases from childhood onwards to young adults and reaches a plateau in adulthood. These findings hold true for all human personality dimensions as well as for males and females, irrespective of the method used to measure the traits [143-145]. Thus it seems as if a stable structure of personality is, once it occurs, more or less preserved into old age. These findings fit to our repeatability measurements in mice that increase from adolescent to young adult individuals and remain relatively stable from about 70 days of age onward (see fig. 3). In our dataset the young adolescent individuals did not show repeatable activity patterns, a phenomenon which has also been found in behaviour of other species like three-spined sticklebacks and wild guinea pigs [146,147]. In a number of species individual stability in traits has been found to appear already early in life [148]. Such emergence of (temporarily) stable individual behavioural trajectories does not rule out the possibility of an increased stabilisation over lifetime. This especially holds true as subjects are usually analysed within relatively short intervals only. One immanent problem in measuring repeatability of behaviour over a lifetime is the enormous change in the behavioural repertoire: A new born, altricial mouse with its particular specialisations (e.g. suckling behaviour) and limitations (e.g. in sensory-motor control) differs widely from the highly differentiated adults of the same species. This problem has also been faced by psychologists studying humans, who found it difficult to assess continuity over the whole period from child- to adulthood due to distinct assessment strategies used at different ages [149]. Consequently, the developmental path over the whole lifetime is not straight forward to measure and might be hard to retrace. Nevertheless, as any behavioural phenotype observed at any point in time is based upon the individual life history, the deciphering of the accumulation of genetic and epigenetic predispositions and individual experiences is of particular importance.

## Conclusions and outlook

This review sheds light on the necessity for careful consideration of individual life histories of experimental subjects like mice and other species. Any investigation using genetically more or less identical inbred mice must consider that such genetic uniformity does not at all translate into phenotypic uniformity. We demonstrated that typically measured behaviours develop over the lifetime, setting the stage for a wealth of gene by environment interactions. There also is good evidence that the emergence of individual difference in itself might be inherent in the developmental process and possibly adaptive and thus cannot be eliminated by standardisation [6,103]. As a side note, variability should not only be considered a nuisance as in correlational analyses greater coefficients of determination are computed if there is more variability among the observations [150].

Individual experiences at certain ages might substantially affect results of behavioural tests administered later in life. If someone is interested in stable and predictable behaviour on an individual level, adult mice might be more suitable as test subjects. On the other hand, if one looks at treatment effects inducing long term behavioural changes, one might prefer to introduce the treatment during early phases of life. Taken together, one should be aware of these confounds and communicate as much information on life history events as possible in the method section of any publication.

What is missing so far are elaborated life-time approaches analysing behavioural development of laboratory mice. There is an abundance of literature describing the behaviour of mice over narrow ranges of time but surprisingly little is known on detailed behavioural development over the lifetime. Our literature survey revealed that indeed a restricted usage of mice for single experiments is the norm. This seems also to be true in experiments that do not include physiological measurements or tissue sampling that would necessarily involve sacrificing the animals. We urge here to preserve mice in future research and conduct follow up studies using mice that already were characterised earlier in their lives. In addition, we opt for analysing behaviour on an individual basis with special emphasis on stability of measures taken at different ages. Given the relatively short lifespan of the species this endeavour can be realised within a reasonable time-frame.

## Authors’ contributions

VB, PMS, and LL wrote the manuscript. LL supervised the experiments and analysed the data presented in the case studies. All authors read and approved the final version of the manuscript.

## Declaration

The Publication of this paper was funded by the German Research Foundation (DFG-Forschergruppe FOR 1232) and the Open Access Publication Fund of Bielefeld and Muenster University.

## Competing interests

The authors declare that they have no competing interests.
